# Building protein-protein interaction networks for *Leishmania* species through protein structural information

**DOI:** 10.1186/s12859-018-2105-6

**Published:** 2018-03-06

**Authors:** Crhisllane Rafaele dos Santos Vasconcelos, Túlio de Lima Campos, Antonio Mauro Rezende

**Affiliations:** 1 Microbiology Department of Instituto Aggeu Magalhães – FIOCRUZ, Recife, PE Brazil; 2 Bioinformatics Plataform of Instituto Aggeu Magalhães – FIOCRUZ, Recife, PE Brazil; 3 Genetics Department of Universidade Federal de Pernambuco, Recife, PE Brazil

## Abstract

**Background:**

Systematic analysis of a parasite interactome is a key approach to understand different biological processes. It makes possible to elucidate disease mechanisms, to predict protein functions and to select promising targets for drug development. Currently, several approaches for protein interaction prediction for non-model species incorporate only small fractions of the entire proteomes and their interactions. Based on this perspective, this study presents an integration of computational methodologies, protein network predictions and comparative analysis of the protozoan species *Leishmania braziliensis* and *Leishmania infantum*. These parasites cause Leishmaniasis, a worldwide distributed and neglected disease, with limited treatment options using currently available drugs.

**Results:**

The predicted interactions were obtained from a meta-approach, applying rigid body docking tests and template-based docking on protein structures predicted by different comparative modeling techniques. In addition, we trained a machine-learning algorithm (Gradient Boosting) using docking information performed on a curated set of positive and negative protein interaction data. Our final model obtained an AUC = 0.88, with recall = 0.69, specificity = 0.88 and precision = 0.83. Using this approach, it was possible to confidently predict 681 protein structures and 6198 protein interactions for *L. braziliensis*, and 708 protein structures and 7391 protein interactions for *L. infantum*. The predicted networks were integrated to protein interaction data already available, analyzed using several topological features and used to classify proteins as essential for network stability.

**Conclusions:**

The present study allowed to demonstrate the importance of integrating different methodologies of interaction prediction to increase the coverage of the protein interaction of the studied protocols, besides it made available protein structures and interactions not previously reported.

**Electronic supplementary material:**

The online version of this article (10.1186/s12859-018-2105-6) contains supplementary material, which is available to authorized users.

## Background

Leishmaniasis represents a series of infections that have as etiological agents species of parasites of the genus *Leishmania*. Belonging to the group of neglected tropical diseases, with more than 90 endemic countries and approximately 1 million new cases per year, leishmaniasis has become a worldwide public health problem [1]. Despite efforts to develop vaccines and new drugs against these diseases, no effective vaccine has been made available, and existent drugs have serious limitations on their use, such as high toxicity, resistant parasites selected by drug pressure and incompatible costs in countries underdeveloped [2–4].

Observing the number of reported cases of leishmaniasis and the difficulties in the treatment and prevention, it is clear the need for approaches that allow a wider understanding of the mechanisms of the diseases, and then we will be able to accelerate the steps toward the development of new drugs. It is already known that comprehension about interactions between proteins and the behavior of this biological system are key information to achieve that goal [5–7], and once this data is obtained in ‘omics’ scale, it allows the prediction of biological function [8–11], identification of changes at gene expression regulation associated with a disease [6, 12], identification of major modules and essential proteins associated [6, 13]. In the end, the analysis of this data generates critical information for the development of new specific drugs, also making possible to predict side effects of new drugs and to understand the side effects of drugs already used [14–16].

Several methodologies, capable of handling and generating large-scale protein interaction data, have been employed, such as the experimental techniques of yeast two-hybrid and affinity purification coupled with mass spectrometry [17]. However, because the problems involving experimental methods, such as cost, laboriousness and susceptibility to systemic errors, over the years, several computational methods have been developed and used to predict protein interaction networks (PIN) [18, 19].

The computational methods can be categorized in different approaches: compiling existing data available in the literature, named text mining [20], data prediction methods based on primary-structure, evolution and tertiary-structure, such as the methods by sequence homology [21–23], co-location [24], similarity of phylogenetic distribution [25] and rigid-body docking [26–28]. Thus, applying bioinformatics tools, extracting and manipulate biological information have been possible to predict protein interaction networks quickly, efficiently and generally with satisfactory numbers of nodes and interactions [6].

Protein interaction networks have been used in some studies with the objective of selecting promising therapeutic targets [29–31], and the protein interaction data contained in this type of network has already been used in the pharmaceutical industry to development of new drugs [32]. Despite the most of the studies involving protein interaction data embraced by the pharmaceutical industry are concentrated in the area of oncology, this breakthrough highlights the value of information contained in a PIN, and it encourages researchers to obtain such data in other areas, like infectious diseases, where analyses using PINs have already been carried out for *Mycobacterium tuberculosis* [33], *Plasmodium falciparum* [34], and *Brugia malayi* [35], which are agents that cause tuberculosis, malaria and filariasis, respectively.

PIN analysis is one of the most promising methodologies for identifying therapeutic targets, understanding drug action and predicting side effects [36]. The use of this approach to the development of new drugs for leishmaniasis is possible, but few data of protein interaction for *Leishmania* species are available. Large-scale experimental methodologies have been used, but they have been directed to host-leishmania interaction [37], so most of the available networks were obtained by computational methods such as PIN predicted through sequence similarity [38, 39] and those predicted through text mining, co-occurrence and co-expression deposited in the String database [40]. However, despite the multiple methodologies used, less than 50% of the proteome of the *Leishmania* species are present in these PINs.

Due to the limited data available on protein interaction for species of *Leishmania*, and considering the importance of this information to accelerate the steps for development of new drugs, we predict here a PIN for *Leishmania braziliensis* and *Leishmania infantum* using physical interaction data between protein structures. It is worth to mention those two species were selected as they belong to two distinct subgenera, *Viannia* and *Leishmania*, respectively, and they are the main leishmania pathogens in Brazil [41, 42], causing mainly cutaneous and muco-cutaneous disease (*Viannia*) and visceral disease (*Leishmania*). Therefore, a meta approach [43, 44] that combines two different methods of predicting PIN was applied: the rigid-body method, that predicts interaction through an exhaustive search of orientations of a protein in relation to the other one based on its atomic coordinates; and the template-based method, that use structural similarity between proteins and known protein complexes [28]. This methodology has not yet been used for *Leishmania* proteomes, hence it allows a complementation for existent available networks, providing new information on interactions and inserting new proteins into these networks. At the end, it is possible to improve and increase the possibilities of data extraction for selection of potential new drug targets.

## Methods

### Prediction of protein structures

The sequences of the predict proteomes of *L. braziliensis* and *L. infantum* version 8.0 were obtained from the TriTrypDB database [45]. The use of computational methods to predict three-dimensional conformation of the proteins was necessary because just few structures for those proteomes were deposited in the Protein Data Bank (PDB) [46]. To perform this task, we applied template-based protein structural modeling methodologies through the Modeller [47] version 9.14 and Modpipe version 2.2.0 [48] algorithm packages, and the Mholline [49] and Protein Homology/analogy Recognition Engine version 2.0 (Phyre2) [50] web-servers.

The modeling algorithm of the Modeller package (model-single) predicts three-dimensional models from the comparative modeling using the alignment of the target sequence against the template sequence, and extracting the spatial constraints from the atomic coordinate file of the template, obeying the terms of a probability density function based on empirical data [47]. The templates were selected using the specific protein alignment algorithm (blastp) of the Basic Local Alignment Search Tool (BLAST) package [51], which made possible to analyze the sequence identity and coverage alignment of the leishmania proteomes against the data deposited in the PDB. Only templates with a minimum of 50% identity and 80% coverage were used. Afterward, two tools were used to perform the Modeller input alignment between the target and template sequences. First, the algorithm for alignment of the modeller package (align2d) [47], and second, the Mafft tool version 7.0 [52]. Align2d is based on dynamic programming algorithm [53], and it takes into account the atomic coordinates of the template [47]. In contrast, Mafft is based on Fast Fourier Transform, and it uses iterative refinement that takes into account evolutionary information to generate alignment [52]. Both alignments were used to predict three-dimensional structures. Modpipe is an automated version of the Modeller package, and it was used to enable a different template search applying profile-profile and sequence-profile alignment [48].

The Mholline server also uses the modeling algorithm of the Modeller package, but it uses the Blast Automatic Targeting for Structures (BATS) and Filter tools to evaluate the quality of the templates, and then to select the best template for comparative modeling [49].

Unlike the tools already mentioned, the Phyre2 server has its own structural modeling algorithm, which implements ab-initio modeling for the portion of the protein which no template has been found. In addition, Phyre2 selects templates based on alignment of Hidden Markov Models via HHsearch [50, 54].

In general, the available template-based protein modeling tools can efficiently predict protein structures when they are executed with high quality templates and identity values between query and template proteins are greater than 25% [55]. In addition, for using structures, which have been predicted by these methods, to computational assays of protein interaction, it is often necessary to perform a full-atomic refinement simulation to increase the quality of the models [56, 57]. Therefore, all predicted structures were submitted to the Modrefiner [57] refinement algorithm.

The quality of the models was evaluated against stereochemical and energetic parameters using Procheck [58] tool and against the standard Discrete Optimized Protein Energy (DOPE) function of the Modeller package [59]. The evaluation of these parameters allows checking conformational stability and approximation of the model to the correct folding [60]. Thus, only models that obtained values for these parameters according to the recommendation of the used tools (torsion angles in a more favorable region in ramachandran plot calculated by Procheck > = 90% and normalized DOPE <= − 1) were submitted to computational tests of protein interaction.

### Prediction of protein interactions using docking methods

The protein models were grouped according to the subcellular localization predicted by the Wolfpsort tool [61], thus reducing the possibility of false positive interaction prediction, besides decreasing the computational time spent on interaction predictions through docking. The three-dimensional protein models of each group were applied to two docking methodologies: first template-based docking through the Prism Protocol [62] tool, and second, the rigid-body docking through the Megadock [63] tool version 4.0.2.

The Prism Protocol requires as input atomic coordinates of two proteins, and a template set formed by pairs of proteins that are known to interact. This tool applies the Naccess [64] and Multiprot [65] softwares to compare the residues responsible for the interaction in the template set with the surface residues of a pair of target proteins, and then Prism Protocol uses this information to infer interaction between a pair of target proteins. In the end, a predicted protein complex is subjected to flexible refinement and energy minimization using the Fiberdock [66] tool [62]. The generated complexes are ranked according to the global energy binding score, and they are selected if they have a score equal to or less than 0. This threshold is the same one used by the developers of the tool to predict interactions in the Prism Web Server [67].

In parallel, the Megadock tool uses only the atomic coordinates of two proteins, and considering shape complementarity, electrostatic and hydrophobic interactions, it computes a set of interaction solutions for a candidate pair of proteins [63]. The prediction of protein interaction through the de novo docking methodology, like Megadock applies, can be described as a binary classification problem, where the resulting set represents a possible or non-possible interaction. To perform this classification, we first used two algorithms based on clustering for evaluating the docking solutions, the Megadock package clustering algorithm [63, 68] and the Calibur tool [69]. The first one generates an affinity value for a predicted Protein-Protein Interaction (ppi-score), this value takes into account the similarity between the solutions and the z-score of the docking score [63, 68], while the second tool groups the solutions by Root Mean Square Deviation (RMSD), and it finds a suitable distance for that grouping, which we call here Calibur-score. This distance is then used to infer whether this interaction represents a true interaction or does not [69]. These scores were submitted to machine learning algorithms in order to classify the complexes generated by rigid-body method.

### Prediction of protein interactions using machine-learning techniques

Initially, we obtained a benchmark data set for the construction of machine-learning predictors of protein interactions. To do so, all the steps performed by the de novo docking were also applied to a set of positive interaction data, composed of 119 protein pairs that are known to interact, obtained from the Benchmark 4.0 database [70], and to a set of negative interaction data, composed of 147 non-interacting protein pairs obtained from the Negatome database [71]. Hence, our final training/test dataset was composed by 266 total entries, where the Calibur and PPI-scores were used as feature inputs, and the outputs were set as “1” for interacting protein pairs, and “0” for the non-interacting pairs. The construction of the learning models was performed using R (https://cran.r-project.org) along with the following libraries: *stats* (Linear Regression Model), *e1071* (Support-Vector Machine and Naive Bayes), *randomForest* (Random Forest), *neuralnet* (Neural Network/Perceptron) and *gbm* (Gradient Boosting Method). For performance assessment and visualization, we used *ROCR*, *PRROC*, *ggplot2* and *plotly* packages.

Six popular machine-learning algorithms for binary classification were trained with default parameters. We performed 100 training/test iterations where we randomly selected 70% of the positive and 70% of the negative interaction data, using them as training sets for each model, then we used the remaining 30% as test sets, calculating the accuracy and area under the curve (AUC) of the Receiver Operating Characteristic (ROC) graph. In addition, Precision and Recall values were calculated for each iteration. We highlight that it was not part of the present work to exhaustively find optimum parameters for each machine-learning method used. After all iterations, we generated boxplots showing the AUCs for each model, and performed statistical tests (pairwise t-tests and TukeyHSD) comparing the performance across the different algorithms. Finally, the model that presented the best performance was selected to classify *Leishmania* interaction data.

The best models built based on the training sets generated response values, ranging from 0 to 1, for the interaction prediction of each pair of proteins. Precision, recall and specificity values were analyzed to define a response value threshold to classify the positive or negative interaction controls. Following the *Leishmania* predictions, protein pairs with response values above this threshold were selected and used as input for Cytoscape for network visualization and topological analysis.

### Topological analysis and selection of essential proteins for the network

Most of the biological networks present free-scale topology, that is, the distribution of the number of connections for each node (degree) follows a power law, where there are few network components (nodes) with a high degree and many network components with a low degree [72]. This feature is strongly related to the stability of the networks, as it makes them resistant to random attacks [72–74].

Other properties of biological networks are their clustering tendency, which can be reflected by the Clustering Coefficient (CC), and their small world effect, caused by having a small number of steps separating any two components of the network, which can be evaluated through the Mean Shortest Path (MSP) [74]. The evaluation of these properties in the network allows validating the data, considering that these characteristics are different from random networks [74, 75].

Thus, we used the Cytoscape software along with the Network Analyzer plugin to evaluate the networks produced based on the free-scale model proposed by Barabasi and Oltvai [10]. The CC and MSP were also compared to 1000 random networks produced by the Random Network plugin (http://apps.cytoscape.org/apps/randomnetworks), and the differences were analyzed through empirical *p-value*.

In addition, to assess the behavior of an interaction network, some topological features can be used to select essential proteins for PIN stability. This is possible due to the relationship between the protein centrality and its role in cell survival [76–78]. In this way, the CytoHubba [79] plugin was used to calculate the Degree Centrality (DC), Betweenness Centrality (BC) and Bottleneck (BN) for each protein.

## Results

### Prediction of protein structures

Protein structures were predicted for 31.13 and 31.39% of the *L. braziliensis* and *L. infantum* proteomes, respectively, by at least one of the modeling tools (Table 1). About those sets of predictions, approximately 4% of both proteomes obtained structures with values referring to free energy and stereochemical properties in according to the thresholds recommended by the evaluation tools. With the use of the structural refinement tool, the percentages of accepted models raised to 8.11 and 8.56% for *L. braziliensis* and *L. infantum* proteomes, respectively (Table 1).

**Table 1:** Total protein structure predicted by each program

Species	Total proteome	Align2d/Modeller		Mafft/Modeller		Modpipe		Mholline		Phyre2		Total	
		p.s	a.s	p.s	a.s	p.s	a.s	p.s	a.s	p.s	a.s	p.s	a.s
*L. braziliensis*	8357	518	163	518	165	1529	479	1858	63	1374	259	2604	681
*L. infantum*	8239	512	181	512	176	1579	504	1856	74	1415	255	2587	708

*p.s* total of Predicted Structures

*a.s* total of Accepted Structures (Structures with values referring to free energy and stereochemical properties according to the thresholds determined by the standardized Dope algorithm and by the Procheck tool)

The use of multiple structure prediction tools allowed predicting structures for a reasonable quantity of proteins. Thus, based on the accepted models in according to the thresholds used, and in order to select the most accurate predicted three-dimensional structure, we selected for each protein with more than one accepted model, the predicted structure with the lowest free energy and highest percentage of torsion angles in the most favorable region of the ramachandran plot (Table 2).

**Table 2:** Total protein per tool with lower free energy structure and higher percentage of torsion angles in the most favorable region of the ramachandran plot

Species	Align2d/Modeller	Mafft/Modeller	Modpipe	Mholline	Phyre2	Total
*L. braziliensis*	88	44	336	28	185	681
*L. infantum*	96	47	344	31	190	708

### Performance evaluation of machine learning models

**Fig. 1:**
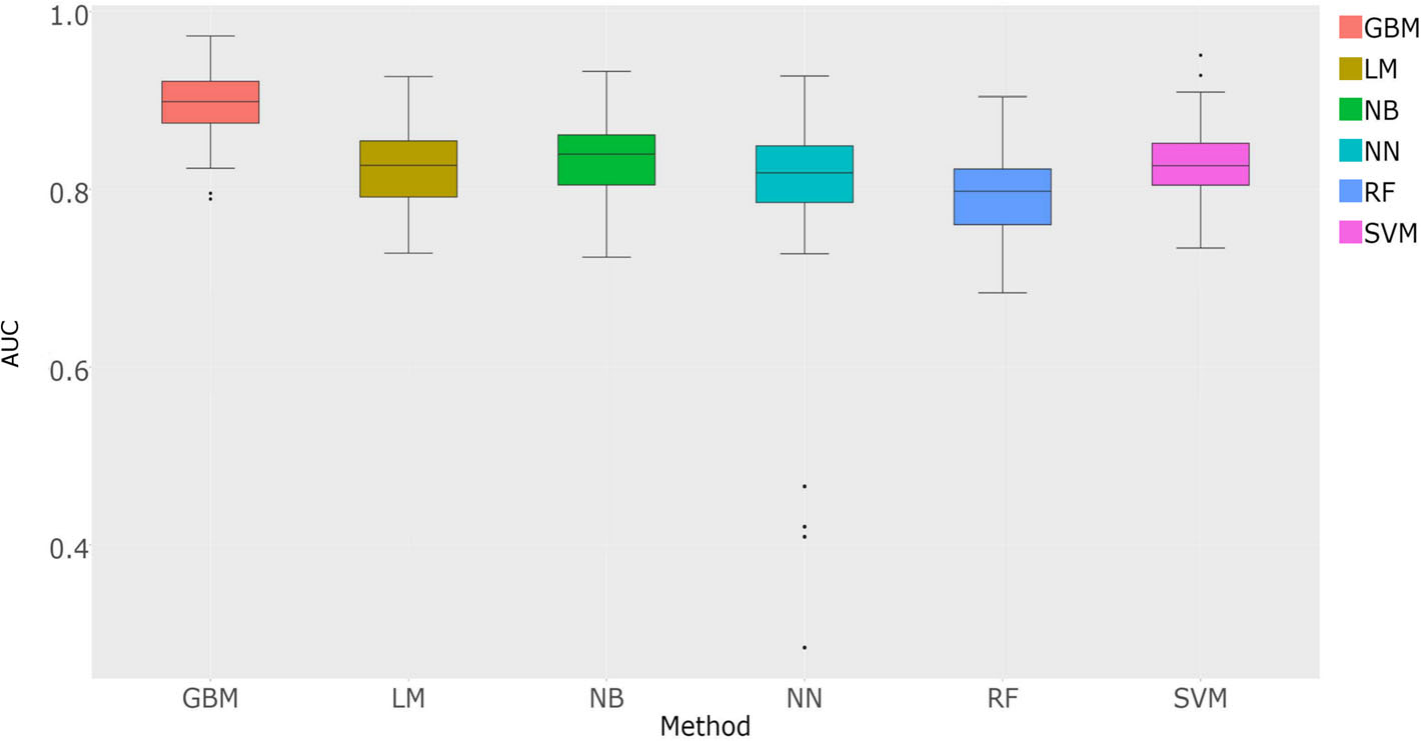
Performance evaluation through the AUC values obtained during the 100 training/tests of machine learning models used to predict interaction between proteins. GBM: Gradient Boosting Method; LM: Linear Regression Model; NB: Native Bayer; NN: Neural Network; RF: Random Forest; SVM: Support Vector Machine

As presented in the methodology section, machine learning algorithms were evaluated against positive and negative interaction datasets used as controls. Based on this analysis, the *gbm* (model available at https://crhisllane.wixsite.com/ppinleishmania) technique showed a better performance when compared to other machine learning algorithms, obtaining an AUC = 0.88 (Fig. 1). We were able to improve the *gbm* model by setting “shrinkage=0.1, n.trees=100, interaction.depth=3, bag.fraction=0.5, train.fraction=0.8, n.minobsinnode=10, cv.folds = 5, class.stratify.cv = TRUE” parameters. The *gbm* algorithm calculates a response value ranging from 0 to 1, for which a minimum threshold of 0.46 has been determined based on controls to indicate interaction between the proteins. This threshold has recall equal to 0.69, specificity equal to 0.88 and precision equal to 0.83.

The use of the response value generated by *gbm* model to evaluate the outcome of the interactions also showed a higher performance when compared to the analysis using only the ppi-score, which obtained an AUC of 0.72, recall equal to 0.65 and precision equal to 0.68. Even when we compared to other studies that used the same interaction prediction tools [28, 63], our recall and precision of response value were higher.

### Prediction of protein interaction

The interaction prediction was performed between proteins that shared the same cell compartment (Table 3). Two proteins of *L. infantum* (LinJ.30.2360, LinJ.31.2540) were the only ones classified in the Peroxisome and Golgi locations, respectively. In this way, they did not share location with any other protein incorporated in the study, making the interaction test impossible, and it was necessary to exclude them from the study. Proteins that had more than one cellular location were maintained in more than one group. In this way, groups of proteins were submitted to the two techniques of interaction prediction using docking, resulting in 82.494 and 88.055 tested interactions by rigid-body method for *L. braziliensis* and *L. infantum*, respectively. Of these, 19.808 and 21.029 interactions were also tested through the template-based method.

**Table 3:** Total proteins in each cell compartment predicted by the Wolfpsort tool

Species	cytoskeleton	cytosol	endoplasmic reticulum	extracellular	mitochondria	nuclear	plasma membrane
*L. braziliensis*	23	351	5	66	150	138	17
*L. infantum*	19	389	2	62	141	134	20

As previously stated, interactions predicted by the template-based method were classified as potential interactions when the global energy binding score was less than or equal to 0. For the interactions predicted by the body-rigid method, due to the amount of solutions generated for each pair of proteins (10,800 solutions), we used clustering tools from which ppi-score and calibur-score were obtained. These values were then submitted to the machine learning algorithm model *gbm*, and the interactions with a response value equal to or greater than 0.46 were described as potential interaction. It worth to remind that *gbm* training model and the threshold of response value were defined based on two sets of experimentally solved protein structures; one set of proteins known to be interacting and one set of non-interacting proteins. Therefore, the leishmania protein interaction predictions are based on validated data (See Methods section).

**Fig. 2:**
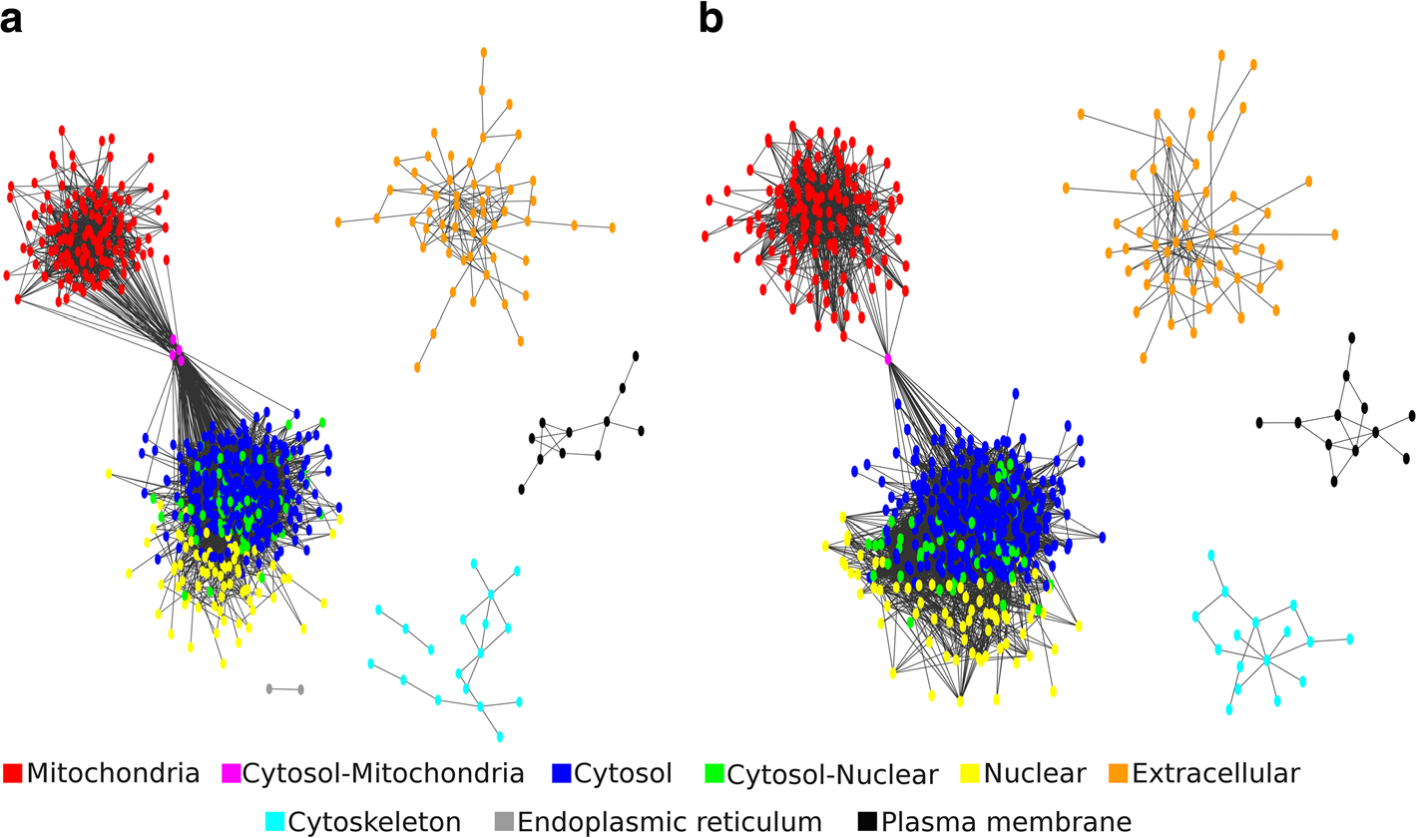
Protein-Protein Interaction Network using Cytoscape 3.5.1. **a** Network for *L. braziliensis*. **b** Network for *L. infantum*. The networks were colored according to the subcellular location

To predict a highly precise interaction network, we apply a meta-approach, using the consensus between both docking methodologies, as proposed by Ohue et al. [28]. Following this methodology, only interactions described as possible by both methodologies were used to build the protein networks (Table 4) (Fig. 2) (Additional files 1 and 2). It is understandable that true positives can be lost applying this meta-approach, but our main goal here was the reduction of potential false positives, thereby increasing the quality of the protein interaction networks generated.

**Table 4:** Interactions described as possible by each tool and consensus

Species	Megadock	Prism	Consensus
*L. braziliensis*	56,520	9216	6198
*L. infantum*	64,163	10,032	7391

A protein network is characterized by a graph composed of nodes representing the proteins and the edges representing the physical interactions between proteins. The networks predicted here had their quality assessed through comparison against 1000 random networks, where the values of Clustering Coefficient and Mean Shortest Path were obtained (Table 5). The Clustering Coefficient, which measures the density of interactions close to a protein in the network [80], was significantly higher in the networks of *Leishmania* species than in random networks. The same behavior was observed when the Mean Shortest Path was evaluated. Both measures are related to the robustness of the network, and the comparisons with random networks suggest the predicted networks are compatible with biological networks, and they are not a product of random insertion of interactions.

**Table 5:** Evaluation of the topological characteristics of protein interaction networks predicted through structural information

*L. braziliensis*			
Scale free model	Correlation	*R* ^*2*^	
	0.671	0.795	
Comparison with random networks			
Measure	Predicted network	Random network	*P*-value
Clustering Coefficient	0.212	0.161 ± 0.005	*p* < 0.05
Mean Shortest Path	2.680	2.510 ± 0.007	*p* < 0.05
*L. infantum*			
Scale free model	Correlation	*R* ^*2*^	
	0.751	0.811	
Comparison with random networks			
Measure	Predicted network	Random network	*P*-value
Clustering Coefficient	0.233	0.169 ± 0.004	*p* < 0.05
Mean Shortest Path	3.000	2.488 ± 0.006	*p* < 0.05

**Fig. 3:**
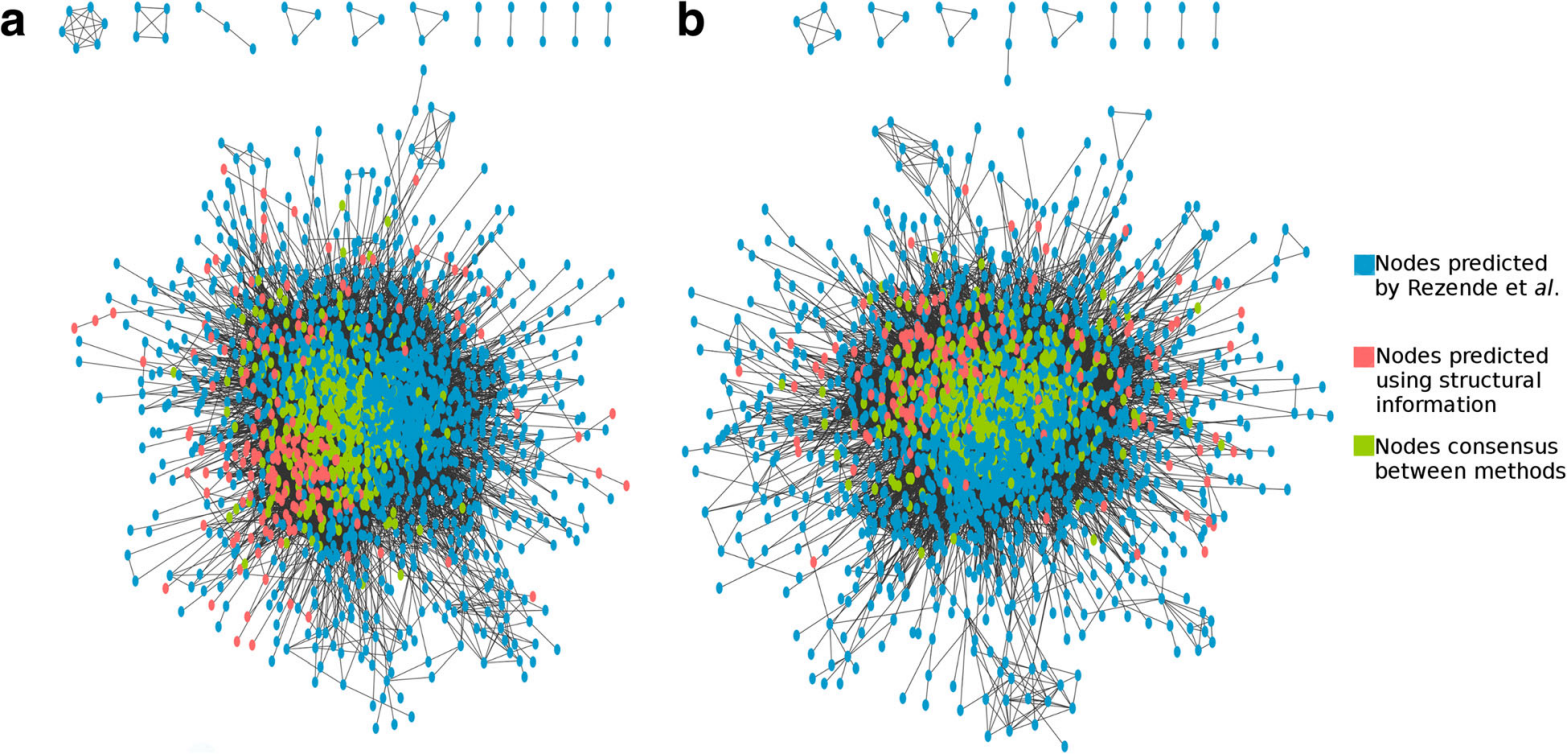
Interaction Protein Networks predicted through structural information adding the networks predicted by Rezende et al. [38]. **a** Network for *L. braziliensis*. **b** Network for *L. infantum*. The networks were colored according with method of prediction interaction used

In order to quantify the new information generated by this methodology and to improve the protein interaction networks of *Leishmania* species, we incorporated the network predicted here with the networks predicted by Rezende and collaborators through the Interolog Mapping method [38] (Fig. 3). The protein interaction networks, resulting from the merging of the networks predicted by both methodologies, continued to present a behavior consistent with biological networks, and different from random networks (Table 6).

**Table 6:** Evaluation of the topological characteristics of the protein interaction networks predicted through structural information and merged to the networks predicted by Rezende et al. [38]

*L. braziliensis*			
Scale free model	Correlation	*R* ^*2*^	
	0.905	0.832	
Comparison with random networks			
Measure	Predicted network	Random network	*P*-value
Clustering Coefficient	0.381	0.144 ± 0.002	*p* < 0.05
Mean Shortest Path	2.832	2.555 ± 0.003	*p* < 0.05
*L. infantum*			
Scale free model	Correlation	*R* ^*2*^	
	0.917	0.837	
Comparison with random networks			
Measure	Predicted network	Random network	*P*-value
Clustering Coefficient	0.381	0.149 ± 0.002	*p* < 0.05
Mean Shortest Path	2.817	2.537 ± 0.003	*p* < 0.05

With the merged networks, it was possible to verify the use of structural information added 201 and 181 proteins to the network of *L. braziliensis* and *L. infantum*, respectively. In addition, it was possible to predict 6002 interactions for *L. braziliensis* and 7119 interactions for *L. infantum*, which were not obtained by the Interolog Mapping method, increasing the knowledge about the interactomes of these species.

### Topological analysis for selection of essential proteins

**Fig. 4:**
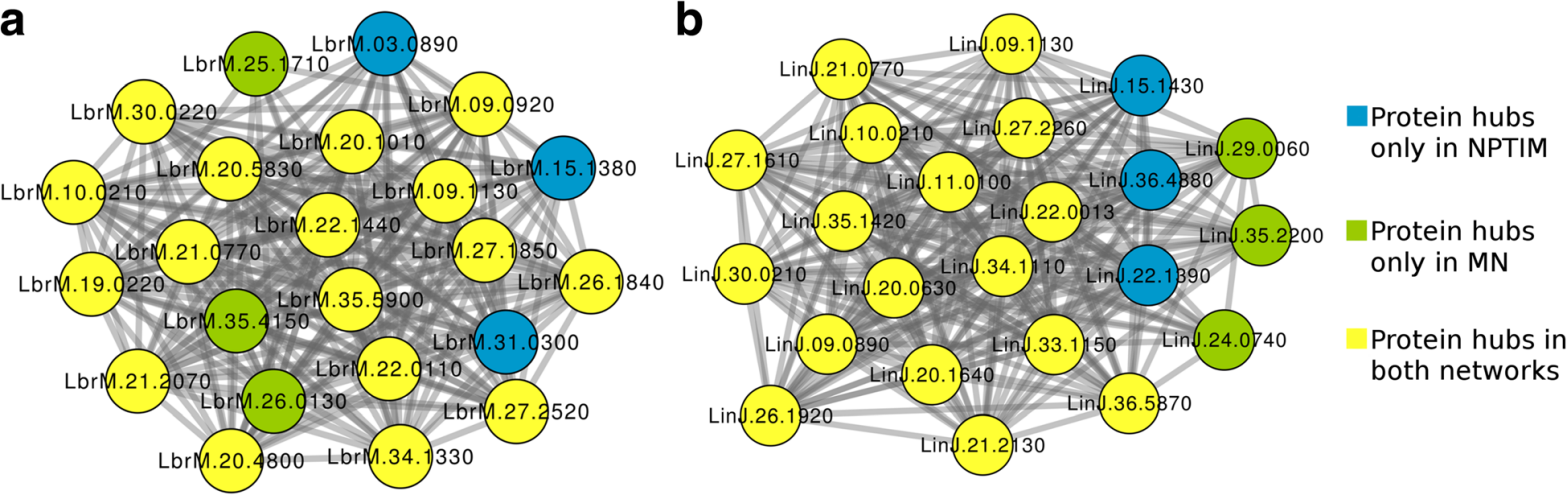
Integration of the sub networks formed by the 20 proteins with the highest Degree of connectivity in predicted protein interaction networks of *L. braziliensis* (**a**) and *L. infantum* (**b**)

The analysis of the topological context of each protein was performed in the predicted networks through structural information (NPTSI) and in the networks predicted through Interolog Mapping (NPTIM) [38], separately, as well in the merged network (MN), resulting from the interaction data obtained in both methodologies. The topological index local-based method Degree was calculated for all the proteins present in the networks (Additional file 3), being possible to select the 20 proteins with the highest number of direct interactions with neighbor proteins (Fig. 4).

Through the degree of connectivity, it was possible to observe that the insertion of new proteins and interactions forming MN did not significantly alter the list of hub proteins presented in the NPTIM (Fig. 4), since the 3 proteins that were substituted among the 20 most connected proteins, remained between the 25 most connected proteins in MN (Additional file 3).

**Fig. 5:**
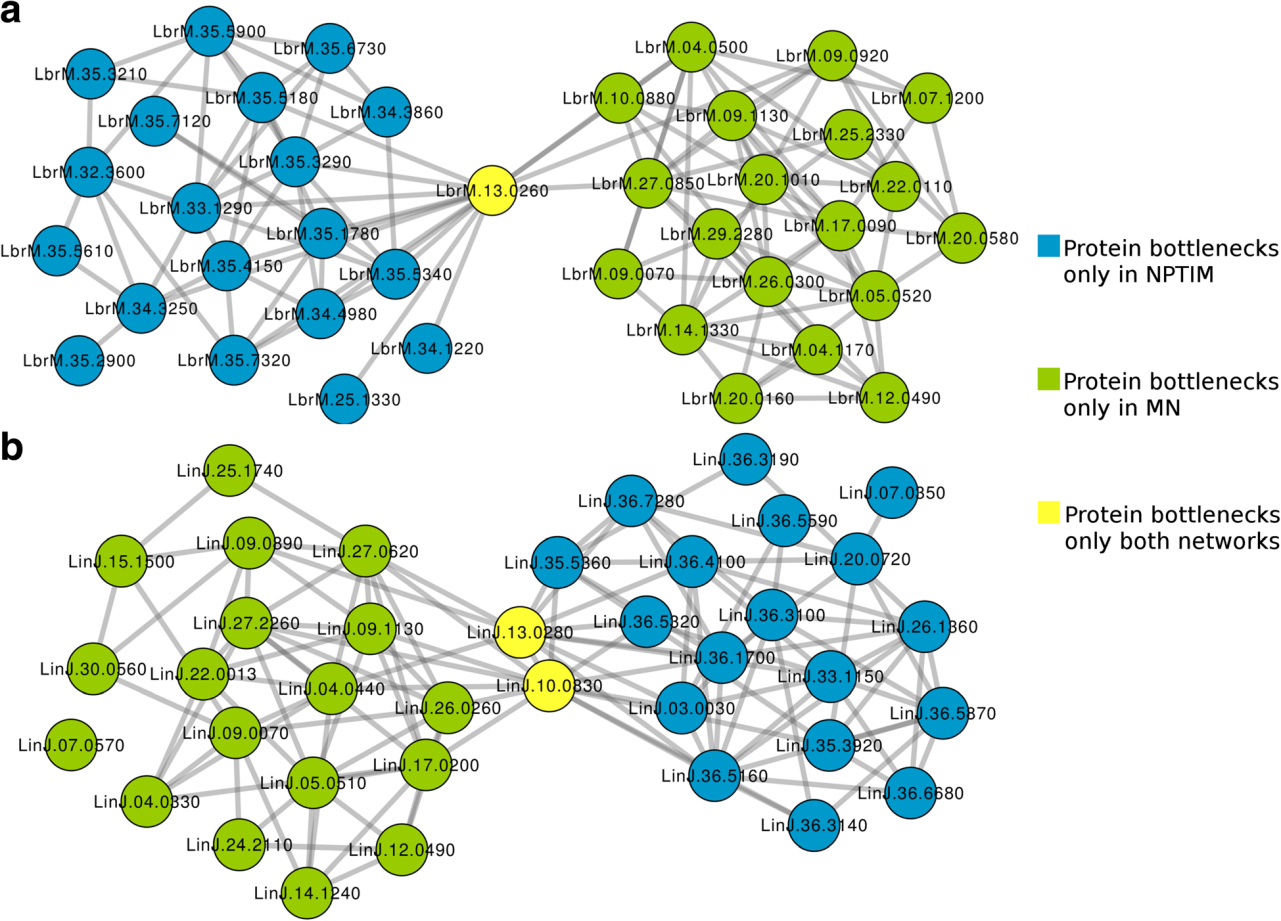
Integration of the sub networks formed by the 20 proteins with the highest value of Bottlenecks in predicted protein interaction networks of *L. braziliensis* (**a**) and *L. infantum* (**b**)

**Fig. 6:**
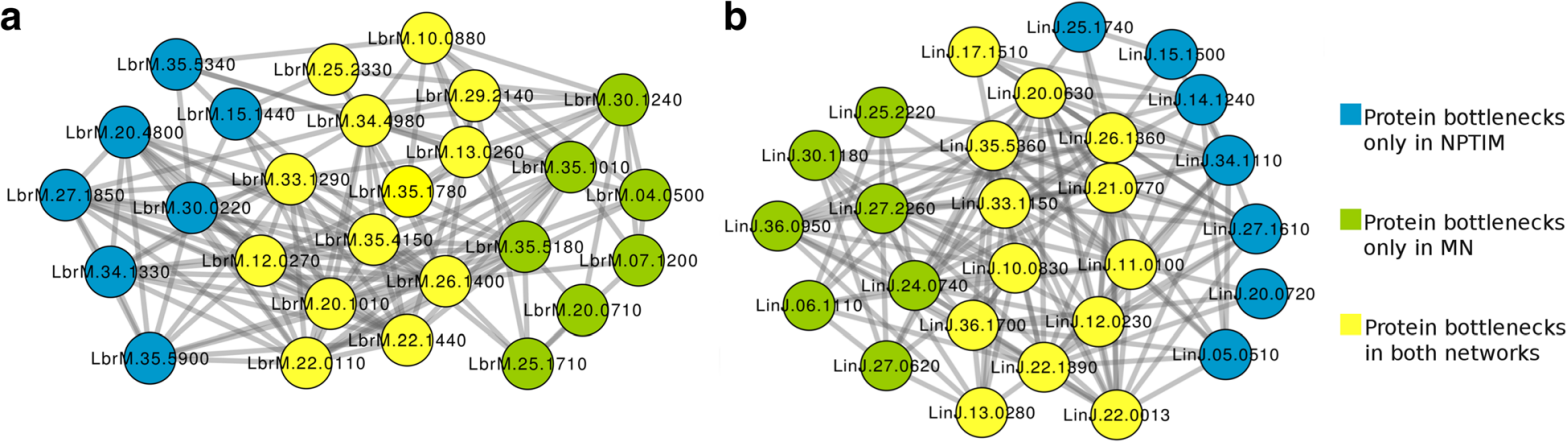
Integration of the sub networks formed by the 20 proteins with the highest value of Betweenness Centrality in predicted protein interaction networks of *L. braziliensis* (**a**) and *L. infantum* (**b**)

Global-based methods were also used to evaluate the topological context of each protein considering the shortest path. For this, the metrics BottleNeck and Betweenness Centrality were calculated for all proteins in the networks (Additional files 4 and 5). Obtaining such values allowed us to observe that, in contrast to the Degree, the insertion of new information into PINs changed drastically the list of bottlenecks proteins (Figs. 5 and 6).

From the evaluation of both metrics of global centrality in MN, it was possible to consider the consensus proteins between the metrics, that is, the bottlenecks proteins selected from the calculations of BC and BN, being these a total of 8 bottlenecks proteins (LbrM.04.0500; LbrM.07.1200; LbrM.10.0880; LbrM.13.0260; LbrM.20.0710; LbrM.20.1010; LbrM.22.0110; LbrM.25.2330) among the 20 evaluated by each metric in the *L. braziliensis* network and 5 bottlenecks proteins (LinJ.10.0830; LinJ.13.0280; LinJ.22.0013; LinJ.27.0620; LinJ.27.2260) in the *L. infantum* network.

The search for the intersection between the nodes selected by all the evaluated metrics (BN, BC and DC) allowed to identify the proteins that present local and global centrality characteristics, being these (LbrM.20.1010 and LbrM.22.0110) in the *L. braziliensis* network and (LinJ.22.0013 and LinJ.27.2260) in the *L. infantum* network. Proteins with this level of centrality were described by Han Jing-Dong and collaborators as “date hubs”. These ones are responsible for the dynamics of the networks, since they are related to the ability of a protein to interact with different proteins at different times [81].

## Discussion

*L. braziliensis* and *L. infantum* are the main species causing leishmaniasis in Brazil. They belong to different subgenera (*Viania* and *Leishmania*, respectively) defined by Lainson and Shaw [82]. Therefore they present some evolutionary differences which can be observed on the clinical disease they can cause. Those differences can be described as the presence of retrovirus in *Viannia* subgenus, which can reflect in the metastatic ability of *L. braziliensis* [83], the different profiles of aneuploidy for both subgenera, which provide a different number of chromosome copy, and can be related with genes expression regulation [84] and drug resistance [85]. Therefore, not just because both species are important pathogens in Brazil, they were also selected here because they can illustrate the difference between the subgenera in the context of protein interactions.

The prediction of protein interaction network based on structural information is a very challenging process, especially when it is applied to species with little structural information obtained experimentally. This is the current reality of *L. braziliensis* and *L. infantum* that at the Uniprot [86] database have only 7 and 10 proteins with available structures, respectively. However, the increasing availability of different computational tools has enabled the protein structural prediction in large-scale, which allowed this study to provide a promising number of predicted protein structures for *Leishmania* species.

Even using a set of parameter values to guarantee models with high quality, we know models might be different from native structure of their proteins. However, as it has been demonstrated by several studies [87–92], the comparative modeling used in this study and the use of sequence similarity are methodologies that provide relevant information for prediction of protein interaction, and they are a helpful alternative approaches to structural biology, being able to provide structural representatives for a large amount of unresolved structure proteins, as it was seen for the data obtained for leishmania.

Obtaining three-dimensional (3D) structures for *Leishmania* proteins opens a parallel path for functional prediction and discovery of new potential targets for drugs based on structural features [93, 94]. This is possible because the function conservation is strictly associated with conservation of the 3D structure [95]. In addition, the availability of these structures allows a search for druggable regions that can be used to design new drugs. Furthermore, with the protein interaction information, it is possible to identify if the druggable regions are part of protein interaction interfaces, and therefore, they can be used to interrupt a protein interaction, and causing damage in the parasite.

Among all the possibilities that can be reached from obtaining protein structures, the prediction of interaction networks provides invaluable structural details for understanding several biological processes [96]. This applicability was first used in 2006 by Kim, P. M. et al. where it was possible to identify structural characteristics of interaction in hubs proteins that could not be identified by methodologies based on sequence [97]. However, even experimental techniques for determination of protein interaction on a large scale are subject to systematic errors and may produce false positives. Similarly, computational docking methods are sometimes unable to distinguish incompatible complexes [27]. To reduce the possibility of errors, we employed machine learning models, trained using positive and negative controls of high confidence. As a result, we obtained a significant difference of AUC, recall and precision when compared to the use of only the affinity values produced by the docking tool. The performance of the *gbm* model was also superior even when compared to the meta-approach applied by Ohue et al. [28].

The classification of interactions as true performed by the *gbm* model produced a network for each species of *Leishmania* addressed in this study. Both networks were evaluated against their topological characteristics, where it was possible to verify their robustness, features compatible with biological networks, and important differences between predicted networks and random networks. One of the characteristics found was the free-scale nature (Table 5), caused by the presence of many proteins that perform few interactions and few proteins, denominated hubs, which perform many interactions. However, this characteristic was improved when the networks predicted through the docking method were incorporated into the networks predicted through the Interolog Mapping method (Table 6). This behavior could be observed because the network constructed here represents only a subset of the true interactome, and as demonstrated by Stumpf and collaborators, the prediction of a network from a small subset can cause a significant deviation of the power law [98].

This free-scale nature of a PIN, as addressed in the Methods section, is strongly related to resilience of network, allowing it to withstand random attacks. This resilience is owed to the fact that the majority of proteins present into an interaction network perform few interactions, thus if they were knockdown, the impact could be not be so strong. However, this same feature makes the network vulnerable to targeted attacks to hub proteins, which are essentials for network stability [99], because they perform a big number of interaction, and we know if we knockdown them, the organism will suffer a great impact. These proteins with higher degree of connectivity are important for cell survival because their essentiality for the transmission of intra-protein information [100, 101]. Therefore, attacking those proteins can destabilize the network causing a break in the transmission of information [36, 101], and hence, the description of such type of protein is an advantage to select targets to drug development.

The selection of hub proteins within the interaction networks was performed through Degree centrality, and as expected, the NPTSI analysis presented different set of protein hubs from those obtained from NPTIM and MN for both species (Additional file 3). This divergence is caused by the difference of protein universe contained in the compared networks*.* However, this behavior was not observed when compared to NPTIM and MN hub proteins, indicating that the insertion of new proteins in the NPTIM did not significantly alter their set of protein hubs (Fig. 4). This result is consistent with the preferred attachment model observed in biological networks [102]. This principle reports that proteins inserted into a real network tend to interact with proteins that already have a higher connectivity degree [103].

The preferential attachment phenomenon is extremely important for the evolutionary process of biological networks, since this process is resulting from the presence of highly conserved domains in hub proteins, and it is related to the free-scale behavior of the networks [102, 104]. The high degree of connectivity makes hub proteins essential for network maintenance, presenting a lethal phenotype upon removal of the protein [105]. This result would be attractive for the development of drugs, however, the degree of conservation of these proteins with host proteins increases the risk of side effects, and may be this is one of the reasons of why they are not the majority of targets already used by the pharmaceutical industry [36]. This can be observed with the analysis of selected date hub proteins, which in UNIPROT showed more than 90% coverage with *Homo sapiens* proteins making difficult or even impossible to use them as targets (data not showed).

On the other hand, the use of random proteins as targets for drugs is not a plausible reality, since the deletion of non-hub proteins, for the most part, has no great impact on the phenotype [105]. As an alternative to this, some studies report that proteins that bind to protein hubs, as well as those with behavior of bottlenecks, which are proteins that alone are responsible for inter-modular interaction, are also promising targets for drugs [106–108]. Proteins with such behavior were recovered from the network using the topological indices BN and BC, generating subnetworks composed of bottlenecks (Figs. 5 and 6). From this analysis, it is possible to observe that the insertion of new proteins and interactions in the networks was able to change the list of proteins that behave like bottlenecks, contrasting with the analysis of the Degree of centrality. It is interesting to notice all analyses presented on Figs. 4, 5 and 6 showed a significant overlap between orthologs from *L. braziliensis* and *L. infantum*. This suggests the conservation of important common process in both species. In addition, this fact can be helpful in order to have the same drug molecule to treat more than one leishmania species.

Our findings are also consistent in favoring central proteins in a global context, since the orthologs proteins LbrM.13.0260 and LinJ.13.0280 reported as nonhubs-bottlenecks have low alignment coverage against STK36 protein ortholog in *H. sapiens*. These results are consistent with the use of this topological feature to select proteins for drug design.

## Conclusion

From the data generated in this study, it is possible to perceive the importance of the use of multiple methodologies to be able to get the interactome of these species. This need is observed even to protein interaction data of most commonly studied species, such as *Homo sapiens*, that still presents a small coverage in front of the complete proteome [109]. Here, we use a methodology not yet applied to the proteome of *Leishmania* species and we describe a binary classification system that was capable of recognizing non-possible complexes. Our findings increase the knowledge in the structural context and the interactome of the species addressed, opening possibilities for future studies in the development or redirection of drugs.

## Additional files

Additional file 1 Protein interaction network of *Leishmania braziliensis* predicted using structural information through the Docking methodology. (XLSX 145 kb)

Additional file 2 Protein interaction network of *Leishmania infantum* predicted using structural information through the Docking methodology. (XLSX 187 kb)

Additional file 3 Degree of connectivity of all proteins present in the networks used in this study. (XLSX 212 kb)

Additional file 4 Value of the Bottleneck topological index of all proteins present in the networks used in this study. (XLSX 207 kb)

Additional file 5 Value of the Betweenness Centrality topological index of all proteins present in the networks used in this study. (XLSX 290 kb)
